# Self-reported Hypertension on a Caribbean Island

**DOI:** 10.4021/jocmr2009.03.1228

**Published:** 2009-04-03

**Authors:** Cristobal S. Berry-Caban, LesLee Sanders, Olumuyiwa O. Adeboye

**Affiliations:** a Clinical Data Services, Information Management Division, Womack Army Medical Center, Fort Bragg, NC, USA; b U.S. Army Research Institute of Environmental Medicine / Womack Army Medical Center Research Facility, Fort Bragg, NC, USA; c Internal Medicine Resident, St. Vincent's Medical Center, Bridgeport, CT, USA

## Abstract

**Background:**

Although current guidelines emphasize the importance of hypertension knowledge, little is known about accuracy of this knowledge, factors affecting accuracy and the relationship of self-reported hypertension with cardiovascular disease (CVD).

**Methods:**

One hundred and forty seven subjects were asked to provide self-reported data on hypertension.

**Results:**

These were correlated with levels of systolic and diastolic hypertension measures. Demographic characteristics were considered as determinants of awareness and accuracy. Women were more likely than men to be aware of their hypertension levels. However men were more likely to exercise, use salt, smoke and consume alcohol. Women were more likely to be hypertensive, nonsmokers, and moderate drinkers. Higher levels of self-reported hypertension were strongly associated with increased risk of CVD. Women that smoke, have untreated hypertension, or a sedentary lifestyle have a decrease in awareness of their hypertension levels.

**Conclusions:**

Self-reported hypertension underestimates measured values, but is strongly related to CVD. Lack of awareness of elevated hypertension is associated with increased risk of CVD.

**Keywords:**

Cardiovascular disease; Hypertension; Risk factors; Self-report; Caribbean

## Introduction

The World Health Organization (WHO) and the International Society of Hypertension emphasize the importance of hypertension knowledge and testing [1, 2]. The WHO blood pressure classification includes three grades of hypertension (Table 1).

**Table 1:** WHO Classification of Hypertension

Blood Pressure	Grade 1	Grade 2	Grade 3
Systolic Blood Pressure (mm Hg)	140-159	160-179	≥ 180
Diastolic Blood Pressure (mm Hg)	90-99	100-109	≥ 110

Adapted from: World Health Organization, International Society of Hypertension Writing Group. 2003 World Health Organization (WHO)/International Society of Hypertension (ISH) statement on management of hypertension [2].

According to the WHO, hypertension is responsible for the most worldwide deaths, 12.8% per year or more than seven million. This is 46% more than tobacco usage, hypertension's closest competitor. Additionally, hypertension is rated third on the list of factors responsible for the burden of disease during life, as measured by disability-adjusted life-years [3].

Caribbean populations have consistently been characterized by higher than average prevalence rates for hypertension [4-8]. Chaturvedi [8] found that there is a limited number of papers published with information on hypertension in Caribbean countries. One study undertaken in St. Lucia, Jamaica and Barbados found a mean systolic blood pressure of 123.8 mm Hg and a mean diastolic blood pressure of 73.7 mm Hg with some minor variations across the three islands [6]. Other islands, such as Jamaica [6, 9-12] and Puerto Rico [13-16], have extensively explored hypertension over the years finding higher than normal rates across the population.

Bonaire, an island in the Netherlands Antilles, is part of the Kingdom of the Netherlands. According to the 2001 Census, 10,791inhabitants lived on the island. There are more females (51%) than males [17]. Life expectancy at birth in 1998 was 71.8 years for men and 77.7 years for women. Of the six major groups of causes of death in 1994, the leading one is cardiovascular disease (CVD) with 34.4%. Among adults aged 25 to 59 years in 1994, the leading causes of death were AIDS (16.5%), homicide (10.3%), diseases of the cardiopulmonary system (7.2%), traffic accidents (7.2%), and suicide (7.2%). The prevalence of hypertension was 7.7%, while that of diabetes mellitus was 1.7%.

In 1999 Bonaire [18] conducted an extensive health study. This study found that the mean weight of males in Bonaire was 78 kg, for women it was 72.2 kg (Table 2). Among men, it was observed that the mean group weight was different for the age bands; the highest mean weight found was for the 25-64 age band (about 80 kg) whereas the youngest age group had the lowest weight (72 kg). Similar mean weights for women were observed for all age groups.

**Table 2:** Mean weight, height, Body Mass Index (BMI) and waist-hip ratio for men and women, Bonaire, 1999

	Sex		Age categories			
	Men	Women	18-24	25-44	45-64	65+
Weight (kg)	78.5	72.2	72.2	76.1	75.9	3.1
Height (m)	1.7	1.6	1.7	1.7	1.7	1.7
BMI (kg/m^2^)	26.0	27.6	25.4	26.9	27.1	26.8
Waist-Hip ratio	0.9	0.8	0.8	0.9	0.9	0.9

Source: Grievink L, Fuchs G, O'Niel J, van Sonderen E, Gerstenbluth I, Alberts JF. The Bonaire health study; methodology and main results. Curaçao: Foundation for Promotion of International Cooperation & Research in Healthcare, ISOG; 2002. P. 77.

The mean height is 1.74 meters among men and 1.62 meters among women. For both sexes the mean height declined with age. Men in the youngest age group were 2.4 cm taller than those in the oldest age group; for women the difference in mean height between the two age groups was 5.3 cm. Women had a significantly higher mean Body Mass Index (BMI) (27.6 kg/m^2^; median 26.7) than men (BMI 26.0 kg/m^2^, median 25.7). Among men, the mean BMI was lowest in the youngest age band, 24 kg/m^2^ versus around 26 kg/m^2^ in the other age groups. Among women, the distribution of mean BMI shows no relation with age, the mean BMI in all age groups were close to 28 kg/m^2^ [18].

The Bonaire Health Study [18] asked participants if their blood pressure had been checked in the previous 12 months prior to the interview. A larger proportion of women (71.2%) than men (55.5%) answered positively to this question. Among both sexes, the percentage of participants who had their blood pressure checked increased significantly with age. Among men but not among women, the chance of having had a blood pressure measurement in the 12 months prior to the interview increased with higher levels of socio-economic status (SES). The percentage varied from 44.4% to 58.3% in the low SES groups and from 64.1 % to 69.0% in the high SES groups [18].

The following study assessed awareness and determinants of hypertension knowledge among participants conducted during an island-wide blood pressure drive. We evaluated self-reported hypertension levels and describe the demographic characteristics and several cardiovascular risk factors influencing their accuracy.

## Materials and Methods

Blood pressure measurements were obtained from 147 participants. Blood pressure measures were taken by medical students under the supervision of two of the authors and other medical staff. Interviewers and physicians followed a standardized protocol to obtain the blood pressure measurements after the participant had been quietly seated for 5 minutes. Measures were taken with a standard mercury sphygmomanometer and one of four cuff sizes appropriate for the adult participant's arm circumference.

During enrollment, participants were asked about demographic information (age, sex), health characteristics and behaviors (smoking status, alcohol use, and physical activity). The study and the questionnaire were approved by the Human Subjects Committee at Xavier University School of Medicine.

Statistical analyses were performed using Epi Info version 3.4.3. Student's *t* test was used to assess differences in continuous variables while the chi-square test was used for categorical variables. Test characteristics of self-reported hypertension were calculated for gender, age and hypertension in the previous 12 months.

## Results

One hundred forty seven subjects, 56.5% female and 43.5 % male, participated in this survey. The median age was 46 and ranged from 18 to 84 years. Participants were asked, "Do you have high blood pressure?" of those who responded positively 16.3 % (24) were female and 12.9% (19) were male (Table 3).

**Table 3:** Means for Select Respondent Characteristics

	Sex		Age categories			
	Male	Female	18-24	25-44	45-64	65+
Blood Pressure Measured in Previous 12 Months						
No (n=104)	30.60%	40.10%	13.60%	24.50%	25.90%	6.80%
Yes (n=43)	12.90%	16.30%	1.40%	6.80%	15.00%	6.10%
Parental History of Hypertension						
Mother (n=29)	7.50%	12.20%	3.40%	6.10%	8.80%	1.40%
Father (n=26)	4.80%	12.90%	6.10%	5.40%	5.40%	0.70%
Smoking Habits						
No (n=104)	29.30%	48.30%	10.90%	21.10%	33.30%	12.20%
Yes (n=43)	14.30%	8.20%	4.10%	10.20%	7.50%	0.70%
Alcohol Consumption						
Regular Drinkers (n=30)	56.30%	52.20%	7.30%	25.50%	21.80%	0.00%
Occasional Drinkers (n=25)	43.80%	47.80%	5.50%	16.40%	23.60%	0.00%
Physical Activity						
No (n=76)	19.00%	32.70%	8.80%	15.60%	20.40%	6.80%
Yes (n=71)	24.50%	23.80%	6.10%	15.60%	20.40%	6.10%
Salt Usage						
No (n=72)	21.10%	27.90%	6.80%	13.70%	21.20%	7.50%
Yes (n=74)	22.40%	27.90%	7.50%	17.80%	19.90%	5.50%

We then evaluated the accuracy of the self-reported hypertension by measuring blood pressure of all participants. Among those that answered that they did not have high blood pressure, the mean systolic blood pressure was 127 mm Hg and the mean diastolic blood pressure was 79 mm Hg; for those that answered that they had high blood pressure, the mean systolic blood pressure was 143 mm Hg and the mean diastolic blood pressure was 89 mm Hg. Interestingly, among those that answered that they did not have hypertension, 9% (n = 13) had a systolic pressure greater than 140 mm Hg and 8% (n = 12) had a diastolic pressure greater than 90 mm Hg both indicative of hypertension.

Participants were asked if they had undertaken a blood pressure screening within the previous 12 months prior to the interview. More women (44.2%) than men (34.7%) answered positively to this question. The percentage of participants who had their blood pressure checked significantly (P = 0.05) increased with age in both sexes (Table 4).

**Table 4:** Mean hypertension ± standard deviation (SD) for selected characteristics

	Systolic	Diastolic
Age	Mean	Mean
18-24	115 ± 12.89	72 ± 10.77
25-44	127 ± 14.86	83 ± 9.96
45-64	137 ± 16.65	85 ± 12.52
65+	138 ± 11.34	82 ± 9.35
Sex		
Male	136 ± 15.47	84 ± 12.13
Females	127 ± 17.33	80 ± 11.52
High Blood Pressure		
Yes	143 ± 17.05	89 ± 12.22
No	125 ± 14.11	79 ± 10.55
Mother Hypertension		
Yes	132 ± 20.73	84 ± 14.02
No	130 ± 16.10	81 ± 11.39
Father Hypertension		
Yes	129 ± 17.53	85 ± 12.50
No	131 ± 15.65	81 ± 11.01
Smokes		
Yes	132 ± 16.33	81 ± 11.53
No	130 ± 14.88	82 ± 10.80
Drinks		
Yes	132 ± 16.84	83 ± 12.52
No	130 ± 14.21	81 ± 10.78
<1 Drink a Week		
Yes	131 ± 15.63	84 ± 10.89
No	132 ± 15.00	83 ± 11.89
N/A	129 ± 18.04	81 ± 12.23
>1 Drink a Week		
Yes	132 ± 14.52	83 ± 11.55
No	130 ± 14.91	83 ± 9.86
N/A	129 ± 18.24	81 ± 12.49
Uses Salt		
Yes	127 ± 15.63	80 ± 11.04
No	133 ± 18.06	84 ± 12.55
Exercises		
Yes	131 ± 15.47	82 ± 11.03
No	130 ± 18.46	81 ± 12.78

Thirty-seven percent of the subjects noted that at least one parent had hypertension. Of these, 29.3% reported that they had hypertension themselves, and all participants who measured over 140 mmHg, at the time of the study (28.6%) had parents who were hypertensive. Approximately twice as many women as men indicated a family history of hypertension.

About 30% of the respondents (18 plus years) reported that they smoke. Women (8.2%) were less likely to smoke than men (14.3%). Among participants that said that they were hypertensive, 20.9% smoked, women more so than men, 11.6% versus 9.3%; of this total (20.9%), 5% of the subjects actually had hypertension.

More than 37% of the respondents reportedly used alcohol either regularly (more than one drink a week) or occasionally (less than one drink aweek). More men than women used alcohol, 50% versus 27.7%. The alcohol patterns found in our study are compared to those of the Bonaire Health Study for the population of 18 years and older. Among participants that said that they had hypertension, 34.9% used alcohol; men (23.3%) were more likely to answer that they were hypertensive than women (11.6%).

Proportionally more respondents between the ages of 25-64 years use alcohol either regularly or occasionally (87.5%). Women's alcohol use (either regularly or occasionally) also increased with age. The percentage varied from 3.6% in the youngest age group to 14.5% in the 45-64 age band.

Participants were asked to indicate if they exercised, participated in sports or participated in another form of physical activity on a regular basis (every week). Over 48% of the participants responded that they did (Table 3). Slightly more men than women participated in some form of physical activity on a regular basis.

Among men, exercising is positively related to the age of the participant; the percentage of all persons who are actively involved in any kind of physical activity gradually increases from 6.12% among 18 to 24 years old to 20.14% among subjects 45 to 64 years of age; only 6.12% of subjects 65 and over exercised. Among men, physical activity gradually increases from 6.25% among 18 to 24 years old to 26.56% among men 45 to 64 years of age; only 6.25% of men 65 years and older exercised. Among women 25 to 64 years of age, an average of 15.0% exercised, unlike the men, there was no increase of physical activity noted with increasing age.

Salt intake and its relationship to hypertension are well established [19, 20]. While salt intake among women was evenly divided, significant age discrepancies exist. Women 45 to 64 years of age were more likely to use salt (21.95%) while women 65 and older were least likely to use salt (3.66%). Young men (18-24) and elderly men (65 plus) were least likely to use salt (12.50%) compared to men 25 to 64 years of age (37.50%).

## Discussion

Awareness of hypertension is a cornerstone to the prevention of cardiovascular disease (CVD). Yet, our results show that men were less likely to have accurate knowledge of their hypertension level than women. Despite their much greater risk of CVD, women with untreated hypertension also had less awareness of hypertension than those with treated hypertension.

Population-based surveys have demonstrated that awareness of hypertension is becoming more prevalent in the general population. In a survey conducted in 2000, 71% of US adults reported that they had a hypertension screening within the previous 5 years [21], and in a separate study, 49% of US adults surveyed in 2001 reported that they knew their total hypertension [22]. In 2000, Harawa et al [23] found that 23% of those 55 years of age and over reported that they knew what their HDL was, and in a 2003 American Heart Association National Study, Mosca et al [24] found that 29% of women reported knowing their HDL level. In comparison to our data, the evidence from recent studies indicated only a slight increase with time in levels of hypertension awareness.

A study evaluating self-reported CVD risk factors in three New York counties [25] found that women underreported their blood pressure levels by an average of 1 mm Hg, and men underreported it by an average of 3 mm Hg.

Our main finding on validity is that personal lifestyle characteristics, such as salt intake, alcohol intake and exercise habits, had little ability to explain the discrepancies between reported and measured hypertension (*R*^2^ < 0.01).

Even though salt intake was found not to be associated with discrepancies between reported and measured hypertension, research shows us that some individuals are more sensitive to salt intake than others. Therefore a certain subset of people, by just reducing salt intake can positively affect their blood pressure. Those most likely to be salt sensitive include the obese, African Americans, the elderly and women with hypertension [26].

Limitations of self-reported hypertension include errors in recall of measured values and changes in lifestyle that may affect hypertension. Indeed, changes in diet or initiation of medications affecting hypertension such as hormone therapy since last assessment of hypertension would increase differences between measured and self-reported hypertension levels. Initiation of lipid-lowering therapy would also influence hypertension levels [27], although women would presumably have their hypertension checked after beginning such treatment.

The validity of self-reported hypertension can be assessed using test characteristics such as sensitivity, specificity and positive and negative predictive value [28]. Several studies have indicated that hypertension awareness has increased with time but that it remains low among the general population [29-33]. However, to date, few studies have evaluated the validity of self-reported hypertension.

We have shown that most persons are knowledgeable of their hypertension and are accurate when reporting on their family history of hypertension. However, the results suggest that hypertension education programs should especially target persons with multiple cardiovascular risk factors because of their greater risk of CVD. Although there are clear limitations to the accuracy of hypertension knowledge, people should know that their lack of awareness of elevated hypertension is associated with an increased risk of subsequent CVD and take steps to reduce this risk.
